# Very rare electrocardiograph abnormalities in meningitis: a case report

**DOI:** 10.11604/pamj.2020.37.231.23158

**Published:** 2020-11-12

**Authors:** Ali Rida Bah, Zeine El Abasse, Othman Benmallem, Rachida Habbal

**Affiliations:** 1 Department of Cardiology, Ibn Rochd University Hospital Casablanca, Casablanca, Morocco

**Keywords:** Meningitis, Osborn, abnormalities, case report

## Abstract

Osborn waves are produced when the J point deviates from baseline. While there are many known causes of Osborn waves, hypothermia remains the most common. We report electrocardiographic changes with Osborn wave in a 32-year-old woman with fatal meningoencephalitis, potentially reversible, probably non-ischaemic myocardial dysfunction may occur in association with acute non cardiac illnesses, such as brain injuries or severe infections. The mechanisms of the electrocardiography (ECG) abnormalities in this disease are unclear.

## Introduction

Bacterial meningitis is an uncommon life threatening disease with serious complications, including neurological disabilities such as hearing loss, visual impairment, seizures and learning disabilities. The heart, kidneys and adrenal glands might also be involved. Cardiac arrhythmias have been rarely described. We report a woman who developed bradycardia, prolonged QT and Osborn waves while recovering from meningitis.

## Patient and observation

We present the case of a 32-year-old patient who was admitted to intensive care for febrile meningeal syndrome. She had no particular pathological history. On examination, she was sleepy, braycardia at 30 beats/min and hypotension at 85/60 mmHg; the temperature was 39.6°C. She showed no motor deficit or cranial nerve damage on neurological examination with the presence of stiff neck. Cardiac and pulmonary ausculation were normal. Full blood count revealed hyper-leukocytosis of 18.54 x 103/mm^3^ with 85% neutrophils without anemia or thrombocytopenia. C-reactive protein level was 168 mg/l. renal function and calcemia were normal. Ultra-sensitive troponin slightly increased to 33 ng/ml who was not in favor of myocarditis. Examination of the cerebrospinal fluid by lumbar punction showed a protein content of 12 g/l with 590 white cells per mm^3^, predominantly poly-nuclear neutrophils (80%). Glucorachia was 1.3 g/l. There was *Streptococcus agalactiae* (group B) confirmed to bacteriology.

**Figure 1:**
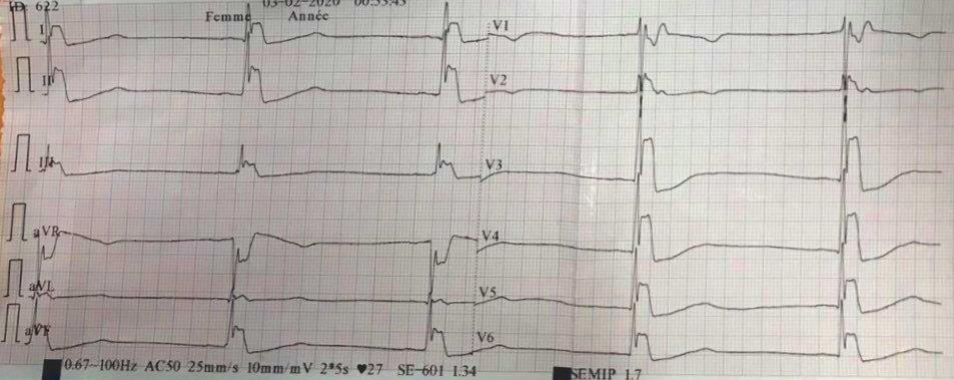
ECG revealed a third degree sinoatrial block with bradycardia at 27 beats/min, QT prolongation and Osborn waves

At admission, a 12-lead electrocardiogram (ECG) revealed a third degree sino-atrial block with bradycardia at 27 beats/min, enlargement of QRS with QT prolongation; ST segment and J point elevation in almost all leads: ST segment changes were convex upwards, camel saddle appearance evocative of Osborn waves (Figure 1). At transthoracic echocardiogram was normal with good ventricular contractility without image of vegetation or pericardial effusion. Chest X-ray and cerebral scanner were normal. The patient in the evening presented a state of shock with alteration of consciousness requiring mechanical ventilation and inotropic support. Despite aggressive supports with exogenous catecholamines, severe cardiovascular collapse persisted causing his death.

## Discussion

The association between a variety of acute, non-cardiac illnesses, predominantly cerebral lesions such as subarachnoid hemorrhage (SAH), thromboembolic stroke, brain tumors, meningitis, meningo-encephalitis and ECG changes, without evidence of myocardial ischaemia, has been described over the past 60 years [1]. Most of the reports include abnormalities of the ST segment, the T wave, the QT interval, as well as disturbances of the cardiac rhythm and conduction system. The ECG changes are usually reversible in survivors, although they may persist for several weeks. The most striking ECG abnormality observed [2] in a series of patients with meningitis was the presence of notched T waves in the precordial leads. ST segment elevation and T wave inversion were less frequent. Peaked P waves, prolonged QT interval and arrhythmias (premature ventricular contractions or atrio-ventricular dissociation) were also observed. Myocarditis and pericarditis are known to occur in association with meningitis and may be the cause of the ECG alterations [3].

Osborn, initially described Osborn waves, or J waves, as the “current of injury” in 1953 as a response to hypothermia in dogs and noted that the amplitude of the J wave was inversely related to body temperature [4]. Since its description it has been associated with several other conditions, even at normothermia. The differential for “normothermic Osborn waves” has evolved over the past several decades and has come to include entities such as benign early repolarization, severe hypercalcaemia, myocardial injury following cardiopulmonary resuscitation, illicit drug overdose, channelopathies and with subarachnoid hemorrhage [5]. There have been no cases reported in the literature regarding this association in the absence of hypothermia. In our patient, biology and echocardiography were not in favor of myocarditis or pericardial effusion. His conductive disorders may be related to his meningitis and it is described in the literature, in addition this concept of “normothermic Osborn waves” little know, in our patience is due to severe sepsis.

## Conclusion

The frequency of ECG changes during bacterial meningitis is probably underestimated. Cardiac monitoring is required throughout the disease course. We believe that these changes might be due to abnormal neural conduction.
